# *N*′-[(5-Methyl-3-oxo-2-phenyl-2,3-dihydro-1*H*-pyrazol-4-yl)(thio­phen-2-yl)methyl­idene]benzohydrazide

**DOI:** 10.1107/S1600536812014821

**Published:** 2012-04-13

**Authors:** Hualing Zhu, Jinhua Zhu, Luxia Bu, Jun Shi, Juan Wang

**Affiliations:** a Department of Basic Science, Tianjin Agricultural College, Tianjin Jinjing Road No. 22, Tianjin 300384, People’s Republic of China; b Meteorological Service Center of Shanxi Province, Taiyuan Xinjian Road No. 65, Taiyuan 030002, People’s Republic of China; c Tianjin Xuanzhen Bio-Medicine and Science Development Co. Ltd, The Third Avenue of TEDA, Tianjin 300457, People’s Republic of China

## Abstract

In the title compound, C_22_H_18_N_4_O_2_S, the seven-membered ring generated by an intra­molecular N—H⋯O hydrogen bond adopts an envelope conformation in both of the two independent mol­ecules in the asymmetric unit. In the crystal, mol­ecules are linked into *C*(9) chains along [100] by N—H⋯O hydrogen bonds. The mol­ecules are also weakly linked by C—H⋯O and C—H⋯N inter­actions, forming dimers with edge-connected *R*_2_^2^(9) rings. The dimers are inter­linked by further weak C—H⋯N hydrogen bonds into chains along [010].

## Related literature

For the biological activity of hydrazones, see: Mahalingam *et al.* (2009 ▶); Kocyigit-Kaymakcioglu *et al.* (2009 ▶); Zhang *et al.* (2007 ▶); Gemma *et al.* (2006 ▶). For uses of hydrazones, see: Gupta *et al.* (2007 ▶). For applications of pyrazolone derivatives, see: Li *et al.* (2000 ▶); Shi *et al.* (2005 ▶); Zhang *et al.* (2008 ▶). For related structures, see: Qiu (2009 ▶); Ren (2009 ▶). 
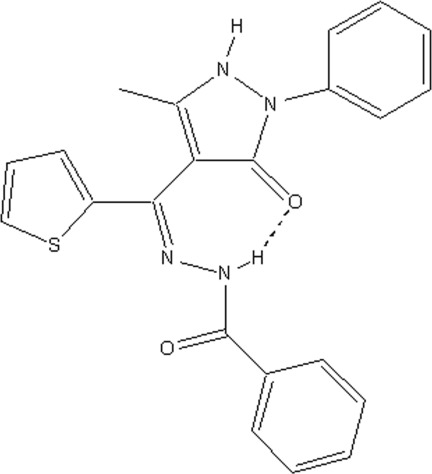


## Experimental

### 

#### Crystal data

C_22_H_18_N_4_O_2_S
*M* *_r_* = 402.46
Orthorhombic, 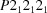

*a* = 13.562 (5) Å
*b* = 16.729 (6) Å
*c* = 17.258 (6) Å
*V* = 3916 (2) Å^3^
*Z* = 8
Mo *K*α radiation
μ = 0.19 mm^−1^
*T* = 113 K
0.20 × 0.18 × 0.12 mm

#### Data collection

Rigaku Saturn724 CCD diffractometer
Absorption correction: multi-scan (*CrystalClear*; Rigaku, 2008 ▶) *T*_min_ = 0.963, *T*_max_ = 0.977
41426 measured reflections
9266 independent reflections
7812 reflections with *I* > 2σ(*I*)
*R*_int_ = 0.082

#### Refinement

*R*[*F* ^2^ > 2σ(*F* ^2^)] = 0.050
*wR*(*F* ^2^) = 0.103
*S* = 1.00
9266 reflections
541 parameters
7 restraints
H atoms treated by a mixture of independent and constrained refinement
Δρ_max_ = 0.53 e Å^−3^
Δρ_min_ = −0.57 e Å^−3^
Absolute structure: Flack (1983 ▶), 4123 Friedel pairs
Flack parameter: −0.01 (6)

### 

Data collection: *CrystalClear* (Rigaku, 2008 ▶); cell refinement: *CrystalClear* ; data reduction: *CrystalClear*; program(s) used to solve structure: *SHELXS97* (Sheldrick, 2008 ▶); program(s) used to refine structure: *SHELXL97* (Sheldrick, 2008 ▶); molecular graphics: *SHELXTL* (Sheldrick, 2008 ▶); software used to prepare material for publication: *CrystalStructure* (Rigaku, 2008 ▶).

## Supplementary Material

Crystal structure: contains datablock(s) I, global. DOI: 10.1107/S1600536812014821/fj2536sup1.cif

Structure factors: contains datablock(s) I. DOI: 10.1107/S1600536812014821/fj2536Isup2.hkl

Supplementary material file. DOI: 10.1107/S1600536812014821/fj2536Isup3.cml

Additional supplementary materials:  crystallographic information; 3D view; checkCIF report

## Figures and Tables

**Table 1:** Hydrogen-bond geometry (Å, °)

*D*—H⋯*A*	*D*—H	H⋯*A*	*D*⋯*A*	*D*—H⋯*A*
N2—H2*A*⋯O2^i^	0.91 (1)	1.79 (1)	2.677 (3)	165 (2)
C15—H15⋯O3^ii^	0.95	2.44	3.383 (3)	171
N6—H6*A*⋯O4^iii^	0.89 (1)	1.80 (1)	2.674 (3)	167 (3)
C36—H36⋯N3^iv^	0.95	2.63	3.326 (3)	131
C14—H14⋯N7^ii^	0.95	2.55	3.322 (3)	139
N4—H4*A*⋯O1	0.91 (1)	1.93 (2)	2.757 (3)	152 (2)
N8—H8*A*⋯O3	0.91 (1)	1.86 (1)	2.715 (3)	157 (2)

Symmetry codes: (i) 
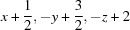
; (ii) 
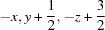
; (iii) 
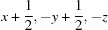
; (iv) 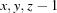.

**Fig. 1.**
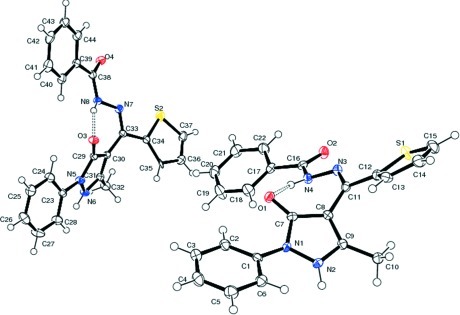
The molecular structure of the title compound with the atom numbering scheme. Displacement ellipsoids are drawn at the 30% probability level. H atoms are presented as a small spheres of arbitrary radii. The hydrogen-bond is shown in dash line.

**Fig. 2.**
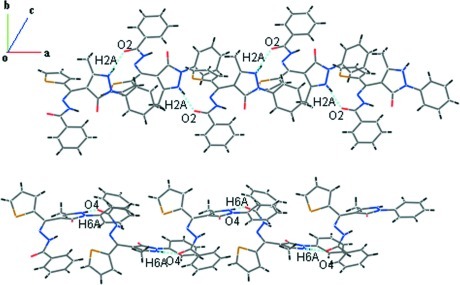
C(9) chains formed by the intermolecular N—H···O hydrogen bonds(shown in dash lines).

**Fig. 3.**
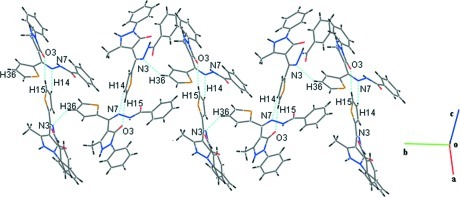
The three-dimensional structure linked by the weak interactions (shown in dash lines)

## supplementary crystallographic information

### Comment

Hydrazones derived from the condensation reactions of hydrazides with aldehydes or ketones show excellent biological properties, such as antimicrobial(Mahalingam *et al.*, 2009), antitubercular(Kocyigit-Kaymakcioglu *et al.*, 2009), anticancer (Zhang *et al.*,2007) and antimalarial (Gemma *et al.*,2006). The hydrazones are also important for their use as plasticizers and stabilizers for polymers, polymerization initiators, antioxidants and as indicators (Gupta *et al.*, 2007). Derivatives of 1-phenyl-3-methyl-4-acyl-5-pyrazolone have found extensive application in coordination chemistry (Shi *et al.*, 2005) and in antibacterial activation (Zhang *et al.*, 2008; Li *et al.*, 2000). Recently, a large number of hydrazone compounds have been reported (Qiu, 2009; Ren *et al.*, 2009). The possible properties and using of hydrazones and the pyrazolone derivatives make it attractive to study these compounds.

The molecular structure of the title compound is shown in Fig. 1. There are two kinds of molecules in the asymmetric unit, which partly differ from each other geometrically. For example, the distance being 1.925 (15) Å between H4A and O1 of the hydrogen bond N4—H4A···O1 in the first kind of molecules is longer than that of the hydrogen bond N8—H8A···O3 in the second kind of molecules which is 1.855 (13) Å. In each independent molecule, the seven-membered ring generated by the intramolecular N—H···O hydrogen bond adopts an envelope conformation. The coplanar atom O1,C7,C8, C11(with the largest deviation of -0.0229 (23) Å for atom C8) and the coplanar atom O3,C29,C30, C33(with the largest deviation of -0.0061 (22) Å for atom C29) form the mean planes of the envelopes, the mean planes and the bonded pyrazole ring are essentially planar, with the dihedral angle of 4.26 (11) ° and 0.56 (9) ° respectively. Plane of N3,N4, H4A and Plane of N7,N8, H8A form the up-warping parts of the envelopes, making a dihedral angle of 61.07 (9)° and 63.20 (98)° with the corresponding mean planes respectively. The pyrazole rings of the two molecules make dihedral angles of 23.16 (8)°, 58.23 (8)°, 31.23 (7)° and 20.06 (7)°, 52.36 (8)°, 20.46 (7)° with the benzene ring of pyrazolone, the thiophen ring and benzene ring of benzoyl hydrazine, respectively.

Two intermolecular N2—H2A···O2 and N6—H6A···O4 hydrogen bonds are observed in the structure(Table1,Fig.2), which link the molecules to form chains of molecules C(9) along [100]. N2 acts as a hydrogen-bond donor to atom O2 at (*x* + 1/2, 1.5 - *y*, 2 - *z*), N6 acts as a hydrogen-bond donor to atom O4 at (*x* + 1/2, 1/2 - *y*, - *z*). Weak C15—H15···O3 and C14—H14···N7 interactions link the molecules to form dimmers with edge-connected *R*_2_^2^(9) rings, the dimmers are interlinked by weak interaction C36–H36···N3 into one dimensional chains along [010] (Table 1, Fig. 3).

### Experimental

The title compound was synthesized by refluxing the mixture of 1-phenyl-3-methyl-4-(2-thenoyl)pyrazolone-5 (30*m* mol) and benzoyl hydrazine (30*m* mol) in ethanol (150 ml) over a steam bath for about 6 h, then the solution was cooled down to room temperature. After seven days, pale yellow block was obtained and dried in air. The product was recrystallized from ethanol which afforded pale yellow crystals suitable for *X*–ray analysis.

### Refinement

During refinement, all H atoms were geometrically positioned and treated as riding on their parent atoms, with C—H = 0.93 Å for the aromatic, 0.96 Å for the methyl and N—H= 0.86 Å with *U*_iso_(H)= 1.2 *U*_eq_(Caromatic, N) or, 1.5*U*_eq_(Cmethyl).

### Crystal data

**Table:**

C_22_H_18_N_4_O_2_S	*F*(000) = 1680
*M**_r_* = 402.46	*D*_x_ = 1.365 Mg m^−^^3^
Orthorhombic, *P*2_1_2_1_2_1_	Mo *K*α radiation, λ = 0.71073 Å
Hall symbol: P 2ac 2ab	Cell parameters from 14107 reflections
*a* = 13.562 (5) Å	θ = 1.7–27.9°
*b* = 16.729 (6) Å	µ = 0.19 mm^−^^1^
*c* = 17.258 (6) Å	*T* = 113 K
*V* = 3916 (2) Å^3^	Prism, colourless
*Z* = 8	0.20 × 0.18 × 0.12 mm

### Data collection

**Table:**

Rigaku Saturn724 CCD diffractometer	9266 independent reflections
Radiation source: fine-focus sealed tube	7812 reflections with *I* > 2σ(*I*)
Graphite monochromator	*R*_int_ = 0.082
Detector resolution: 14.22 pixels mm^-1^	θ_max_ = 27.9°, θ_min_ = 1.7°
ω and φ scans	*h* = −17→17
Absorption correction: multi-scan (*CrystalClear*; Rigaku, 2008)	*k* = −22→22
*T*_min_ = 0.963, *T*_max_ = 0.977	*l* = −22→22
41426 measured reflections	

### Refinement

**Table:**

Refinement on *F*^2^	Secondary atom site location: difference Fourier map
Least-squares matrix: full	Hydrogen site location: inferred from neighbouring sites
*R*[*F*^2^ > 2σ(*F*^2^)] = 0.050	H atoms treated by a mixture of independent and constrained refinement
*wR*(*F*^2^) = 0.103	*w* = 1/[σ^2^(*F*_o_^2^) + (0.0431*P*)^2^] where *P* = (*F*_o_^2^ + 2*F*_c_^2^)/3
*S* = 1.00	(Δ/σ)_max_ < 0.001
9266 reflections	Δρ_max_ = 0.53 e Å^−^^3^
541 parameters	Δρ_min_ = −0.57 e Å^−^^3^
7 restraints	Absolute structure: Flack (1983), 4123 Friedel pairs
Primary atom site location: structure-invariant direct methods	Flack parameter: −0.01 (6)

### Special details

**Table:**

Geometry. All e.s.d.'s (except the e.s.d. in the dihedral angle between two l.s. planes) are estimated using the full covariance matrix. The cell e.s.d.'s are taken into account individually in the estimation of e.s.d.'s in distances, angles and torsion angles; correlations between e.s.d.'s in cell parameters are only used when they are defined by crystal symmetry. An approximate (isotropic) treatment of cell e.s.d.'s is used for estimating e.s.d.'s involving l.s. planes.
Refinement. Refinement of *F*^2^ against ALL reflections. The weighted *R*-factor *wR* and goodness of fit *S* are based on *F*^2^, conventional *R*-factors *R* are based on *F*, with *F* set to zero for negative *F*^2^. The threshold expression of *F*^2^ > σ(*F*^2^) is used only for calculating *R*-factors(gt) *etc*. and is not relevant to the choice of reflections for refinement. *R*-factors based on *F*^2^ are statistically about twice as large as those based on *F*, and *R*- factors based on ALL data will be even larger.

### Fractional atomic coordinates and isotropic or equivalent isotropic displacement parameters (Å^2^)

**Table:**

	*x*	*y*	*z*	*U*_iso_*/*U*_eq_	
S1	−0.03830 (5)	0.71956 (4)	1.11387 (4)	0.02597 (15)	
O1	0.32273 (11)	0.58102 (9)	0.99164 (10)	0.0192 (4)	
O2	0.00489 (12)	0.62704 (10)	0.85706 (10)	0.0225 (4)	
N1	0.41739 (14)	0.68241 (10)	1.04849 (11)	0.0162 (4)	
N2	0.39591 (14)	0.75401 (11)	1.08442 (12)	0.0179 (4)	
N3	0.09446 (14)	0.65442 (11)	0.99061 (11)	0.0175 (4)	
N4	0.14777 (14)	0.63005 (11)	0.92562 (11)	0.0167 (4)	
C1	0.51201 (16)	0.64760 (13)	1.05797 (13)	0.0162 (5)	
C2	0.53891 (17)	0.58191 (13)	1.01313 (14)	0.0208 (5)	
H2	0.4952	0.5606	0.9754	0.025*	
C3	0.63134 (17)	0.54841 (14)	1.02498 (15)	0.0235 (5)	
H3	0.6501	0.5028	0.9957	0.028*	
C4	0.69714 (18)	0.57982 (15)	1.07857 (16)	0.0259 (6)	
H4	0.7604	0.5564	1.0851	0.031*	
C5	0.67008 (18)	0.64501 (15)	1.12212 (15)	0.0262 (6)	
H5	0.7150	0.6671	1.1585	0.031*	
C6	0.57672 (17)	0.67876 (13)	1.11298 (15)	0.0210 (5)	
H6	0.5573	0.7228	1.1442	0.025*	
C7	0.32789 (16)	0.64663 (13)	1.02580 (13)	0.0147 (5)	
C8	0.25253 (16)	0.70038 (13)	1.05077 (13)	0.0153 (5)	
C9	0.29774 (16)	0.76560 (13)	1.08431 (13)	0.0160 (5)	
C11	0.14507 (17)	0.68506 (13)	1.04734 (13)	0.0152 (5)	
C12	0.08755 (17)	0.70277 (13)	1.11724 (14)	0.0182 (5)	
C13	0.12018 (18)	0.70293 (14)	1.19226 (14)	0.0214 (5)	
H13	0.1870	0.6940	1.2063	0.026*	
C14	0.04498 (19)	0.71769 (15)	1.24670 (14)	0.0257 (6)	
H14	0.0558	0.7200	1.3010	0.031*	
C15	−0.0450 (2)	0.72826 (15)	1.21298 (15)	0.0268 (6)	
H15	−0.1040	0.7391	1.2408	0.032*	
C16	0.09352 (17)	0.61021 (13)	0.86222 (13)	0.0165 (5)	
C17	0.14549 (18)	0.56975 (13)	0.79726 (13)	0.0174 (5)	
C18	0.24761 (19)	0.57110 (17)	0.78695 (15)	0.0288 (6)	
H18	0.2883	0.5976	0.8237	0.035*	
C19	0.28974 (19)	0.53406 (18)	0.72339 (17)	0.0339 (7)	
H19	0.3592	0.5360	0.7165	0.041*	
C20	0.23189 (19)	0.49425 (16)	0.66971 (15)	0.0268 (6)	
H20	0.2611	0.4686	0.6263	0.032*	
C21	0.1307 (2)	0.49244 (16)	0.68048 (16)	0.0310 (6)	
H21	0.0902	0.4651	0.6442	0.037*	
C22	0.08813 (19)	0.52985 (15)	0.74315 (16)	0.0274 (6)	
H22	0.0186	0.5283	0.7494	0.033*	
S2	−0.09874 (5)	0.34519 (4)	0.00961 (4)	0.03026 (16)	
O3	0.24984 (11)	0.24796 (9)	0.17686 (9)	0.0199 (4)	
O4	−0.02713 (12)	0.08734 (9)	0.08119 (10)	0.0208 (4)	
N5	0.35661 (14)	0.31492 (11)	0.09300 (11)	0.0186 (4)	
N6	0.34621 (14)	0.35697 (11)	0.02458 (12)	0.0182 (4)	
N7	0.04294 (14)	0.23573 (11)	0.07448 (11)	0.0181 (4)	
N8	0.10040 (14)	0.17233 (11)	0.10211 (12)	0.0170 (4)	
C10	0.25968 (17)	0.84234 (14)	1.11489 (15)	0.0212 (5)	
H10A	0.2638	0.8422	1.1716	0.032*	
H10B	0.1908	0.8491	1.0990	0.032*	
H10C	0.2992	0.8865	1.0943	0.032*	
C23	0.45209 (16)	0.30388 (13)	0.12491 (13)	0.0179 (5)	
C24	0.46974 (18)	0.24164 (14)	0.17687 (15)	0.0227 (5)	
H24	0.4177	0.2074	0.1929	0.027*	
C25	0.56520 (18)	0.23076 (15)	0.20470 (15)	0.0255 (6)	
H25	0.5777	0.1892	0.2409	0.031*	
C26	0.64183 (19)	0.27877 (17)	0.18090 (16)	0.0295 (6)	
H26	0.7068	0.2698	0.1995	0.035*	
C27	0.62270 (18)	0.34085 (17)	0.12900 (16)	0.0305 (6)	
H27	0.6751	0.3740	0.1117	0.037*	
C28	0.52799 (18)	0.35436 (15)	0.10266 (15)	0.0243 (5)	
H28	0.5149	0.3983	0.0693	0.029*	
C29	0.26401 (16)	0.28772 (13)	0.11669 (13)	0.0157 (5)	
C30	0.19616 (16)	0.31593 (12)	0.05886 (13)	0.0159 (5)	
C31	0.25075 (16)	0.35685 (13)	0.00345 (14)	0.0177 (5)	
C32	0.22506 (18)	0.39519 (15)	−0.07270 (14)	0.0233 (5)	
H32A	0.2275	0.4535	−0.0673	0.035*	
H32B	0.1585	0.3789	−0.0882	0.035*	
H32C	0.2724	0.3782	−0.1123	0.035*	
C33	0.08897 (16)	0.30131 (13)	0.05762 (13)	0.0154 (5)	
C34	0.02208 (17)	0.36645 (14)	0.03500 (14)	0.0193 (5)	
C35	0.04202 (17)	0.45161 (14)	0.03426 (14)	0.0204 (5)	
H35	0.1023	0.4764	0.0488	0.025*	
C36	−0.04525 (18)	0.49200 (14)	0.00765 (16)	0.0265 (6)	
H36	−0.0479	0.5483	0.0006	0.032*	
C37	−0.12364 (18)	0.44355 (15)	−0.00669 (17)	0.0288 (6)	
H37	−0.1859	0.4626	−0.0240	0.035*	
C38	0.05203 (17)	0.10249 (13)	0.11298 (14)	0.0174 (5)	
C39	0.09756 (17)	0.04402 (13)	0.16751 (14)	0.0180 (5)	
C40	0.19398 (18)	0.04933 (14)	0.19578 (15)	0.0222 (5)	
H40	0.2371	0.0898	0.1772	0.027*	
C41	0.22648 (19)	−0.00513 (16)	0.25131 (16)	0.0270 (6)	
H41	0.2924	−0.0025	0.2699	0.032*	
C42	0.1631 (2)	−0.06279 (15)	0.27940 (15)	0.0278 (6)	
H42	0.1849	−0.0985	0.3186	0.033*	
C43	0.06866 (19)	−0.06888 (15)	0.25105 (16)	0.0274 (6)	
H43	0.0257	−0.1090	0.2705	0.033*	
C44	0.03588 (19)	−0.01664 (14)	0.19422 (15)	0.0241 (5)	
H44	−0.0286	−0.0222	0.1735	0.029*	
H2A	0.4390 (14)	0.7935 (11)	1.0964 (15)	0.025 (7)*	
H4A	0.2090 (9)	0.6092 (13)	0.9308 (14)	0.016 (6)*	
H6A	0.3952 (14)	0.3734 (15)	−0.0057 (13)	0.034 (8)*	
H8A	0.1528 (13)	0.1844 (15)	0.1327 (13)	0.033 (8)*	

### Atomic displacement parameters (Å^2^)

**Table:**

	*U*^11^	*U*^22^	*U*^33^	*U*^12^	*U*^13^	*U*^23^
S1	0.0178 (3)	0.0374 (4)	0.0227 (3)	0.0044 (3)	0.0035 (3)	0.0032 (3)
O1	0.0183 (9)	0.0169 (8)	0.0223 (9)	0.0009 (6)	−0.0017 (7)	−0.0046 (7)
O2	0.0187 (9)	0.0254 (9)	0.0235 (9)	0.0047 (7)	−0.0045 (7)	−0.0060 (8)
N1	0.0163 (10)	0.0123 (8)	0.0199 (10)	0.0008 (8)	0.0008 (8)	−0.0044 (8)
N2	0.0185 (11)	0.0148 (9)	0.0203 (10)	−0.0024 (8)	0.0002 (9)	−0.0049 (8)
N3	0.0179 (10)	0.0175 (9)	0.0170 (10)	0.0030 (8)	−0.0001 (9)	−0.0010 (8)
N4	0.0147 (11)	0.0190 (10)	0.0163 (10)	0.0014 (8)	−0.0006 (9)	−0.0033 (8)
C1	0.0124 (12)	0.0166 (11)	0.0198 (11)	−0.0010 (9)	0.0032 (9)	0.0032 (10)
C2	0.0197 (13)	0.0227 (12)	0.0199 (12)	−0.0014 (10)	0.0041 (11)	−0.0022 (10)
C3	0.0204 (13)	0.0234 (12)	0.0267 (13)	0.0013 (10)	0.0043 (11)	−0.0017 (11)
C4	0.0186 (13)	0.0299 (13)	0.0291 (14)	0.0069 (11)	−0.0022 (11)	0.0032 (12)
C5	0.0221 (14)	0.0294 (13)	0.0272 (14)	−0.0005 (11)	−0.0046 (11)	0.0003 (12)
C6	0.0235 (13)	0.0187 (11)	0.0207 (12)	0.0003 (10)	0.0000 (11)	0.0009 (10)
C7	0.0163 (12)	0.0151 (10)	0.0126 (10)	−0.0019 (9)	−0.0014 (9)	0.0019 (9)
C8	0.0135 (11)	0.0172 (11)	0.0152 (11)	−0.0008 (9)	0.0011 (9)	0.0008 (9)
C9	0.0156 (12)	0.0168 (11)	0.0157 (11)	0.0013 (9)	−0.0006 (9)	0.0009 (9)
C11	0.0182 (12)	0.0138 (10)	0.0137 (11)	0.0019 (9)	0.0004 (10)	0.0006 (9)
C12	0.0170 (12)	0.0181 (11)	0.0194 (12)	0.0004 (10)	0.0009 (10)	−0.0032 (10)
C13	0.0202 (13)	0.0234 (12)	0.0208 (12)	−0.0058 (10)	0.0013 (10)	0.0006 (10)
C14	0.0313 (15)	0.0274 (13)	0.0183 (12)	−0.0060 (12)	0.0039 (12)	−0.0003 (11)
C15	0.0261 (14)	0.0308 (14)	0.0234 (13)	0.0014 (12)	0.0087 (12)	0.0033 (11)
C16	0.0176 (13)	0.0130 (10)	0.0188 (12)	−0.0009 (10)	−0.0035 (10)	−0.0007 (9)
C17	0.0234 (13)	0.0138 (10)	0.0149 (11)	0.0021 (10)	−0.0025 (10)	−0.0005 (9)
C18	0.0211 (14)	0.0414 (16)	0.0237 (13)	−0.0044 (12)	−0.0027 (11)	−0.0102 (12)
C19	0.0189 (14)	0.0537 (18)	0.0292 (15)	0.0023 (13)	0.0019 (12)	−0.0114 (14)
C20	0.0335 (16)	0.0269 (13)	0.0198 (12)	0.0041 (12)	0.0040 (12)	−0.0064 (11)
C21	0.0347 (16)	0.0319 (14)	0.0264 (14)	−0.0033 (12)	−0.0041 (12)	−0.0153 (12)
C22	0.0196 (13)	0.0320 (14)	0.0307 (14)	−0.0008 (11)	−0.0020 (12)	−0.0090 (12)
S2	0.0233 (3)	0.0276 (3)	0.0399 (4)	0.0031 (3)	−0.0033 (3)	−0.0053 (3)
O3	0.0203 (9)	0.0225 (8)	0.0168 (8)	−0.0038 (7)	−0.0004 (7)	0.0045 (7)
O4	0.0210 (9)	0.0189 (8)	0.0225 (9)	−0.0014 (7)	−0.0076 (8)	0.0038 (7)
N5	0.0202 (11)	0.0189 (9)	0.0167 (10)	−0.0020 (8)	−0.0001 (8)	0.0041 (8)
N6	0.0176 (11)	0.0201 (10)	0.0169 (10)	−0.0022 (9)	0.0010 (9)	0.0053 (8)
N7	0.0186 (10)	0.0165 (9)	0.0192 (10)	0.0018 (8)	−0.0001 (9)	0.0019 (8)
N8	0.0126 (10)	0.0172 (9)	0.0212 (11)	−0.0002 (8)	−0.0031 (9)	0.0035 (8)
C10	0.0185 (12)	0.0201 (11)	0.0249 (12)	0.0003 (10)	0.0010 (11)	−0.0033 (11)
C23	0.0139 (12)	0.0217 (11)	0.0182 (12)	−0.0012 (9)	−0.0004 (10)	−0.0033 (10)
C24	0.0213 (13)	0.0241 (12)	0.0226 (12)	−0.0017 (10)	0.0007 (11)	0.0024 (11)
C25	0.0263 (14)	0.0270 (13)	0.0233 (13)	0.0031 (11)	−0.0037 (11)	0.0013 (11)
C26	0.0165 (13)	0.0416 (15)	0.0304 (15)	0.0012 (12)	−0.0085 (12)	−0.0003 (13)
C27	0.0222 (14)	0.0385 (15)	0.0307 (15)	−0.0127 (12)	−0.0042 (11)	0.0062 (13)
C28	0.0232 (13)	0.0266 (13)	0.0230 (12)	−0.0040 (11)	−0.0049 (11)	0.0036 (11)
C29	0.0142 (12)	0.0161 (10)	0.0167 (11)	−0.0007 (9)	0.0030 (10)	−0.0009 (10)
C30	0.0172 (12)	0.0141 (10)	0.0162 (11)	0.0005 (9)	0.0012 (10)	0.0022 (9)
C31	0.0164 (12)	0.0158 (10)	0.0209 (12)	0.0009 (9)	−0.0002 (10)	0.0002 (10)
C32	0.0222 (13)	0.0273 (13)	0.0204 (13)	0.0001 (11)	−0.0005 (11)	0.0072 (11)
C33	0.0143 (12)	0.0175 (11)	0.0145 (11)	−0.0010 (9)	0.0019 (10)	−0.0001 (9)
C34	0.0186 (9)	0.0220 (10)	0.0173 (11)	0.0011 (9)	0.0032 (9)	−0.0003 (10)
C35	0.0136 (11)	0.0227 (10)	0.0250 (12)	0.0090 (9)	0.0097 (10)	0.0138 (10)
C36	0.0225 (12)	0.0204 (12)	0.0368 (15)	0.0061 (10)	−0.0032 (12)	0.0042 (12)
C37	0.0252 (14)	0.0256 (13)	0.0355 (16)	0.0087 (11)	−0.0043 (12)	−0.0011 (12)
C38	0.0157 (12)	0.0181 (11)	0.0184 (11)	0.0018 (9)	0.0006 (10)	0.0002 (10)
C39	0.0199 (12)	0.0175 (11)	0.0166 (11)	0.0020 (10)	−0.0022 (10)	0.0006 (9)
C40	0.0221 (13)	0.0198 (12)	0.0248 (13)	0.0043 (10)	−0.0035 (11)	−0.0014 (10)
C41	0.0271 (15)	0.0270 (13)	0.0270 (14)	0.0078 (12)	−0.0116 (12)	−0.0071 (12)
C42	0.0383 (16)	0.0216 (12)	0.0235 (13)	0.0111 (12)	−0.0032 (12)	0.0022 (11)
C43	0.0300 (15)	0.0218 (12)	0.0305 (15)	0.0063 (11)	0.0045 (12)	0.0092 (11)
C44	0.0250 (14)	0.0220 (12)	0.0252 (13)	0.0041 (11)	−0.0011 (12)	0.0039 (11)

### Geometric parameters (Å, º)

**Table:**

S1—C15	1.719 (3)	O4—C38	1.232 (3)
S1—C12	1.731 (2)	N5—N6	1.382 (3)
O1—C7	1.248 (3)	N5—C29	1.397 (3)
O2—C16	1.238 (3)	N5—C23	1.419 (3)
N1—N2	1.380 (2)	N6—C31	1.345 (3)
N1—C7	1.409 (3)	N6—H6A	0.889 (10)
N1—C1	1.419 (3)	N7—C33	1.295 (3)
N2—C9	1.346 (3)	N7—N8	1.400 (3)
N2—H2A	0.906 (10)	N8—C38	1.353 (3)
N3—C11	1.301 (3)	N8—H8A	0.908 (10)
N3—N4	1.395 (3)	C10—H10A	0.9800
N4—C16	1.360 (3)	C10—H10B	0.9800
N4—H4A	0.905 (10)	C10—H10C	0.9800
C1—C2	1.393 (3)	C23—C28	1.386 (3)
C1—C6	1.394 (3)	C23—C24	1.395 (3)
C2—C3	1.388 (3)	C24—C25	1.393 (3)
C2—H2	0.9500	C24—H24	0.9500
C3—C4	1.389 (4)	C25—C26	1.376 (4)
C3—H3	0.9500	C25—H25	0.9500
C4—C5	1.374 (4)	C26—C27	1.396 (4)
C4—H4	0.9500	C26—H26	0.9500
C5—C6	1.395 (3)	C27—C28	1.381 (3)
C5—H5	0.9500	C27—H27	0.9500
C6—H6	0.9500	C28—H28	0.9500
C7—C8	1.428 (3)	C29—C30	1.437 (3)
C8—C9	1.379 (3)	C30—C31	1.390 (3)
C8—C11	1.481 (3)	C30—C33	1.474 (3)
C9—C10	1.481 (3)	C31—C32	1.503 (3)
C11—C12	1.467 (3)	C32—H32A	0.9800
C12—C13	1.368 (3)	C32—H32B	0.9800
C13—C14	1.409 (3)	C32—H32C	0.9800
C13—H13	0.9500	C33—C34	1.471 (3)
C14—C15	1.363 (4)	C34—C35	1.450 (3)
C14—H14	0.9500	C35—C36	1.438 (3)
C15—H15	0.9500	C35—H35	0.9500
C16—C17	1.487 (3)	C36—C37	1.360 (4)
C17—C22	1.387 (3)	C36—H36	0.9500
C17—C18	1.397 (3)	C37—H37	0.9500
C18—C19	1.383 (4)	C38—C39	1.491 (3)
C18—H18	0.9500	C39—C44	1.394 (3)
C19—C20	1.385 (4)	C39—C40	1.399 (3)
C19—H19	0.9500	C40—C41	1.394 (3)
C20—C21	1.385 (4)	C40—H40	0.9500
C20—H20	0.9500	C41—C42	1.380 (4)
C21—C22	1.377 (4)	C41—H41	0.9500
C21—H21	0.9500	C42—C43	1.375 (4)
C22—H22	0.9500	C42—H42	0.9500
S2—C37	1.703 (3)	C43—C44	1.387 (3)
S2—C34	1.733 (3)	C43—H43	0.9500
O3—C29	1.248 (3)	C44—H44	0.9500
			
C15—S1—C12	91.84 (13)	N5—N6—H6A	125.7 (18)
N2—N1—C7	108.16 (18)	C33—N7—N8	116.74 (19)
N2—N1—C1	119.69 (19)	C38—N8—N7	115.57 (18)
C7—N1—C1	129.57 (18)	C38—N8—H8A	119.4 (16)
C9—N2—N1	109.47 (18)	N7—N8—H8A	117.7 (16)
C9—N2—H2A	122.2 (15)	C9—C10—H10A	109.5
N1—N2—H2A	126.8 (16)	C9—C10—H10B	109.5
C11—N3—N4	116.53 (19)	H10A—C10—H10B	109.5
C16—N4—N3	115.96 (19)	C9—C10—H10C	109.5
C16—N4—H4A	118.8 (15)	H10A—C10—H10C	109.5
N3—N4—H4A	120.6 (16)	H10B—C10—H10C	109.5
C2—C1—C6	120.6 (2)	C28—C23—C24	120.4 (2)
C2—C1—N1	119.8 (2)	C28—C23—N5	119.4 (2)
C6—C1—N1	119.6 (2)	C24—C23—N5	120.2 (2)
C3—C2—C1	118.2 (2)	C25—C24—C23	118.6 (2)
C3—C2—H2	120.9	C25—C24—H24	120.7
C1—C2—H2	120.9	C23—C24—H24	120.7
C4—C3—C2	121.7 (2)	C26—C25—C24	121.5 (2)
C4—C3—H3	119.1	C26—C25—H25	119.2
C2—C3—H3	119.1	C24—C25—H25	119.2
C5—C4—C3	119.5 (2)	C25—C26—C27	119.0 (2)
C5—C4—H4	120.2	C25—C26—H26	120.5
C3—C4—H4	120.2	C27—C26—H26	120.5
C4—C5—C6	120.1 (2)	C28—C27—C26	120.4 (2)
C4—C5—H5	119.9	C28—C27—H27	119.8
C6—C5—H5	119.9	C26—C27—H27	119.8
C1—C6—C5	119.8 (2)	C27—C28—C23	120.0 (2)
C1—C6—H6	120.1	C27—C28—H28	120.0
C5—C6—H6	120.1	C23—C28—H28	120.0
O1—C7—N1	123.6 (2)	O3—C29—N5	123.7 (2)
O1—C7—C8	131.0 (2)	O3—C29—C30	130.9 (2)
N1—C7—C8	105.38 (18)	N5—C29—C30	105.40 (19)
C9—C8—C7	107.87 (19)	C31—C30—C29	107.4 (2)
C9—C8—C11	126.2 (2)	C31—C30—C33	126.7 (2)
C7—C8—C11	125.7 (2)	C29—C30—C33	125.9 (2)
N2—C9—C8	109.06 (19)	N6—C31—C30	109.1 (2)
N2—C9—C10	118.0 (2)	N6—C31—C32	117.4 (2)
C8—C9—C10	132.9 (2)	C30—C31—C32	133.5 (2)
N3—C11—C12	114.7 (2)	C31—C32—H32A	109.5
N3—C11—C8	128.1 (2)	C31—C32—H32B	109.5
C12—C11—C8	117.09 (19)	H32A—C32—H32B	109.5
C13—C12—C11	127.3 (2)	C31—C32—H32C	109.5
C13—C12—S1	110.52 (18)	H32A—C32—H32C	109.5
C11—C12—S1	121.98 (18)	H32B—C32—H32C	109.5
C12—C13—C14	113.4 (2)	N7—C33—C34	112.9 (2)
C12—C13—H13	123.3	N7—C33—C30	127.8 (2)
C14—C13—H13	123.3	C34—C33—C30	119.29 (19)
C15—C14—C13	112.7 (2)	C35—C34—C33	128.0 (2)
C15—C14—H14	123.6	C35—C34—S2	112.07 (17)
C13—C14—H14	123.6	C33—C34—S2	119.88 (17)
C14—C15—S1	111.5 (2)	C36—C35—C34	108.1 (2)
C14—C15—H15	124.2	C36—C35—H35	125.9
S1—C15—H15	124.2	C34—C35—H35	125.9
O2—C16—N4	121.9 (2)	C37—C36—C35	114.9 (2)
O2—C16—C17	120.6 (2)	C37—C36—H36	122.5
N4—C16—C17	117.5 (2)	C35—C36—H36	122.5
C22—C17—C18	118.6 (2)	C36—C37—S2	113.01 (19)
C22—C17—C16	117.4 (2)	C36—C37—H37	123.5
C18—C17—C16	124.0 (2)	S2—C37—H37	123.5
C19—C18—C17	120.2 (2)	O4—C38—N8	122.6 (2)
C19—C18—H18	119.9	O4—C38—C39	120.5 (2)
C17—C18—H18	119.9	N8—C38—C39	117.0 (2)
C18—C19—C20	120.8 (2)	C44—C39—C40	119.5 (2)
C18—C19—H19	119.6	C44—C39—C38	116.0 (2)
C20—C19—H19	119.6	C40—C39—C38	124.4 (2)
C19—C20—C21	118.8 (2)	C41—C40—C39	119.6 (2)
C19—C20—H20	120.6	C41—C40—H40	120.2
C21—C20—H20	120.6	C39—C40—H40	120.2
C22—C21—C20	120.7 (2)	C42—C41—C40	120.1 (2)
C22—C21—H21	119.6	C42—C41—H41	119.9
C20—C21—H21	119.6	C40—C41—H41	119.9
C21—C22—C17	120.8 (2)	C43—C42—C41	120.5 (2)
C21—C22—H22	119.6	C43—C42—H42	119.8
C17—C22—H22	119.6	C41—C42—H42	119.8
C37—S2—C34	91.78 (12)	C42—C43—C44	120.2 (2)
N6—N5—C29	108.91 (18)	C42—C43—H43	119.9
N6—N5—C23	119.41 (18)	C44—C43—H43	119.9
C29—N5—C23	131.63 (19)	C43—C44—C39	120.0 (2)
C31—N6—N5	109.22 (19)	C43—C44—H44	120.0
C31—N6—H6A	124.1 (18)	C39—C44—H44	120.0
			
C7—N1—N2—C9	−1.0 (2)	C29—N5—N6—C31	−0.6 (2)
C1—N1—N2—C9	−164.5 (2)	C23—N5—N6—C31	177.2 (2)
C11—N3—N4—C16	−169.83 (19)	C33—N7—N8—C38	−174.6 (2)
N2—N1—C1—C2	−170.6 (2)	N6—N5—C23—C28	20.2 (3)
C7—N1—C1—C2	29.8 (3)	C29—N5—C23—C28	−162.6 (2)
N2—N1—C1—C6	10.3 (3)	N6—N5—C23—C24	−158.4 (2)
C7—N1—C1—C6	−149.2 (2)	C29—N5—C23—C24	18.8 (4)
C6—C1—C2—C3	0.2 (3)	C28—C23—C24—C25	−1.0 (4)
N1—C1—C2—C3	−178.8 (2)	N5—C23—C24—C25	177.6 (2)
C1—C2—C3—C4	−1.5 (4)	C23—C24—C25—C26	−1.3 (4)
C2—C3—C4—C5	1.0 (4)	C24—C25—C26—C27	1.5 (4)
C3—C4—C5—C6	0.7 (4)	C25—C26—C27—C28	0.7 (4)
C2—C1—C6—C5	1.4 (3)	C26—C27—C28—C23	−3.0 (4)
N1—C1—C6—C5	−179.6 (2)	C24—C23—C28—C27	3.2 (4)
C4—C5—C6—C1	−1.9 (4)	N5—C23—C28—C27	−175.4 (2)
N2—N1—C7—O1	179.9 (2)	N6—N5—C29—O3	−179.8 (2)
C1—N1—C7—O1	−18.7 (4)	C23—N5—C29—O3	2.8 (4)
N2—N1—C7—C8	−0.5 (2)	N6—N5—C29—C30	−0.2 (2)
C1—N1—C7—C8	160.9 (2)	C23—N5—C29—C30	−177.7 (2)
O1—C7—C8—C9	−178.7 (2)	O3—C29—C30—C31	−179.5 (2)
N1—C7—C8—C9	1.8 (2)	N5—C29—C30—C31	1.0 (2)
O1—C7—C8—C11	6.2 (4)	O3—C29—C30—C33	−1.5 (4)
N1—C7—C8—C11	−173.3 (2)	N5—C29—C30—C33	179.0 (2)
N1—N2—C9—C8	2.2 (3)	N5—N6—C31—C30	1.3 (3)
N1—N2—C9—C10	−175.35 (19)	N5—N6—C31—C32	−176.10 (19)
C7—C8—C9—N2	−2.5 (3)	C29—C30—C31—N6	−1.4 (3)
C11—C8—C9—N2	172.6 (2)	C33—C30—C31—N6	−179.4 (2)
C7—C8—C9—C10	174.5 (2)	C29—C30—C31—C32	175.4 (2)
C11—C8—C9—C10	−10.4 (4)	C33—C30—C31—C32	−2.6 (4)
N4—N3—C11—C12	−174.44 (18)	N8—N7—C33—C34	−175.45 (19)
N4—N3—C11—C8	2.5 (3)	N8—N7—C33—C30	4.6 (3)
C9—C8—C11—N3	141.6 (3)	C31—C30—C33—N7	136.9 (3)
C7—C8—C11—N3	−44.2 (4)	C29—C30—C33—N7	−40.7 (4)
C9—C8—C11—C12	−41.5 (3)	C31—C30—C33—C34	−43.1 (3)
C7—C8—C11—C12	132.7 (2)	C29—C30—C33—C34	139.3 (2)
N3—C11—C12—C13	150.7 (2)	N7—C33—C34—C35	160.4 (2)
C8—C11—C12—C13	−26.6 (3)	C30—C33—C34—C35	−19.6 (4)
N3—C11—C12—S1	−24.2 (3)	N7—C33—C34—S2	−16.1 (3)
C8—C11—C12—S1	158.49 (16)	C30—C33—C34—S2	163.83 (17)
C15—S1—C12—C13	1.0 (2)	C37—S2—C34—C35	2.7 (2)
C15—S1—C12—C11	176.6 (2)	C37—S2—C34—C33	179.8 (2)
C11—C12—C13—C14	−176.3 (2)	C33—C34—C35—C36	179.8 (2)
S1—C12—C13—C14	−1.0 (3)	S2—C34—C35—C36	−3.4 (3)
C12—C13—C14—C15	0.4 (3)	C34—C35—C36—C37	2.6 (3)
C13—C14—C15—S1	0.4 (3)	C35—C36—C37—S2	−0.7 (3)
C12—S1—C15—C14	−0.8 (2)	C34—S2—C37—C36	−1.2 (2)
N3—N4—C16—O2	13.4 (3)	N7—N8—C38—O4	20.2 (3)
N3—N4—C16—C17	−168.81 (18)	N7—N8—C38—C39	−158.3 (2)
O2—C16—C17—C22	−19.8 (3)	O4—C38—C39—C44	−15.3 (3)
N4—C16—C17—C22	162.4 (2)	N8—C38—C39—C44	163.2 (2)
O2—C16—C17—C18	158.8 (2)	O4—C38—C39—C40	168.5 (2)
N4—C16—C17—C18	−19.0 (3)	N8—C38—C39—C40	−13.0 (4)
C22—C17—C18—C19	0.8 (4)	C44—C39—C40—C41	−1.2 (4)
C16—C17—C18—C19	−177.8 (3)	C38—C39—C40—C41	174.9 (2)
C17—C18—C19—C20	−0.9 (4)	C39—C40—C41—C42	−1.4 (4)
C18—C19—C20—C21	0.3 (4)	C40—C41—C42—C43	2.3 (4)
C19—C20—C21—C22	0.3 (4)	C41—C42—C43—C44	−0.4 (4)
C20—C21—C22—C17	−0.4 (4)	C42—C43—C44—C39	−2.2 (4)
C18—C17—C22—C21	−0.1 (4)	C40—C39—C44—C43	3.0 (4)
C16—C17—C22—C21	178.6 (2)	C38—C39—C44—C43	−173.4 (2)

### Hydrogen-bond geometry (Å, º)

**Table:**

*D*—H···*A*	*D*—H	H···*A*	*D*···*A*	*D*—H···*A*
N2—H2*A*···O2^i^	0.91 (1)	1.79 (1)	2.677 (3)	165 (2)
C15—H15···O3^ii^	0.95	2.44	3.383 (3)	171
N6—H6*A*···O4^iii^	0.89 (1)	1.80 (1)	2.674 (3)	167 (3)
C36—H36···N3^iv^	0.95	2.63	3.326 (3)	131
C14—H14···N7^ii^	0.95	2.55	3.322 (3)	139
N4—H4*A*···O1	0.91 (1)	1.93 (2)	2.757 (3)	152 (2)
N8—H8*A*···O3	0.91 (1)	1.86 (1)	2.715 (3)	157 (2)

Symmetry codes: (i) *x*+1/2, −*y*+3/2, −*z*+2; (ii) −*x*, *y*+1/2, −*z*+3/2; (iii) *x*+1/2, −*y*+1/2, −*z*; (iv) *x*, *y*, *z*−1.
